# An ethical analysis of UK drug policy as an example of a criminal justice approach to drugs: a commentary on the short film *Putting UK Drug Policy into Focus*

**DOI:** 10.1186/s12954-020-00434-8

**Published:** 2020-12-09

**Authors:** Adam Holland

**Affiliations:** School of Population Health Sciences, University of Bristol, Bristol, UK

**Keywords:** Advocacy, Decriminalisation, Ethics, Film, Harm reduction, Illicit drugs, Policy, Regulation

## Abstract

**Background:**

Drug-related deaths in the UK are at the highest level on record—the war on drugs has failed. A short film has been produced intended for public and professional audiences featuring academics, representatives of advocacy organisations, police and policymakers outlining the problems with, and highlighting alternative approaches to, UK drug policy. A range of ethical arguments are alluded to, which are distilled here in greater depth for interested viewers and a wider professional and academic readership.

**Main body:**

The war on drugs is seemingly driven by the idea that the consumption of illegal drugs is immoral. However, the meaning ascribed to ‘drug’ in the illicit sense encompasses a vast range of substances with different properties that have as much in common with legal drugs as they do with each other. The only property that distinguishes illegal from legal drugs is their legal status, which rather than being based on an assessment of how dangerous they are has been defined by centuries of socio-political idiosyncrasies. The consequences of criminalising people who use drugs often outweigh the risks they face from drug use, and there is not convincing evidence that this prevents wider drug use or drug-related harm. Additionally, punishing someone as a means, to the end of deterring others from drug use, is ethically problematic. Although criminalising the production of harmful drugs may seem more ethically tenable, it has not reduced the supply of drugs and it precludes effective regulation of the market. Other potential policy approaches are highlighted, which would be ethically preferable to existing punitive policy.

**Conclusion:**

It is not possible to eliminate all drug use and associated harms. The current approach is not only ineffective in preventing drug-related harm but itself directly and indirectly causes incalculable harm to those who use drugs and to wider society. For policymakers to gain the mandate to rationalise drug policy, or to be held accountable if they do not, wider engagement with the electorate is required. It is hoped that this film will encourage at least a few to give pause and reflect on how drug policy might be improved.

## Background

In the UK, drug-related deaths are at the highest level on record [1], accounting in 2017 for more than a third of the drug-related deaths in the European Union [2]. Without the burden of ideology framing its aims in terms of a moral impetus, any other field of public policy similarly marred by failure would be swiftly overhauled. To the readership of this journal the problems with criminal justice focused drug policy, underscored by the rhetoric of the war on drugs may seem so plainly evident that they do not warrant stating. Nonetheless, it persists unabated in the UK and to degrees across the world. The propagation of punitive drug policy may be driven indirectly by the power relations between politicians and the electorate in democratic societies as the incentive of votes, or the threat of their loss, inspires a need to not be seen as being ‘soft on drugs’. Alternatively, it may be driven directly by policymakers; this could be due to their unconscious biases, as it is plainer than ever to see in contemporary politics that power does not preclude politicians from being all too human, or as others have despondently suggested, in some cases this could feasibly represent a conscious effort to further the interests of powerful actors and reinforce socioeconomic inequalities [3]. Whether the war on drugs is spurred on by one, or by a combination of these factors, to create a more just society it is necessary to engage with the public to highlight the problems with drug policy, particularly given the lack of transparency in much of contemporary political decision-making. Only through a shift in the understanding of the electorate will those in positions of power be given the mandate to rationalise drug policy or be held accountable if they do not.

With that in mind, with borrowed equipment and assistance gratefully received from friends and colleagues, I produced the short film *Putting UK Drug Policy into Focus*. In the film, the UK situation is used as an example to explore the problems with a criminal justice focused approach to drugs with the optimistic hope that viewers, both national and international, will be motivated to reflect on what a more ethically sound approach would look like. Fourteen stakeholders kindly agreed to be interviewed or to submit footage for the film including academics, representatives of advocacy organisations, police officers and policymakers. Although the film focuses on the UK, some contributors are from further afield as national policy is contingent on international context, and alternative international practice is highlighted. Contemporary arguments in favour of and against maintaining the status quo in drug policy are examined, rather than the antecedents of the current situation as the socio-political context of the past is not a reason to maintain the policy borne from it in the future; socio-political contexts change as does our understanding of the effects of policy decisions.

A range of ethical arguments is alluded to in the film, which are distilled here in greater depth for interested viewers and a wider professional and academic readership. Admittedly, discussion of the idealistic aims of drug policy is unlikely to directly influence those in power [4] and ethical argumentation alone is not sufficient to change policy; however, it is necessary to ensure the rigorous formulation of imperatives to do so [5]. Both deontological and consequentialist perspectives are explored. Deontological theories maintain that the rightness or wrongness of acts is determined by the nature of the acts themselves, the duties of those performing them and the rights of those affected. Consequentialist theories on the other hand maintain that the rightness or wrongness of acts is determined by their consequences [6]. Section one explores and critiques the idea that using those drugs that are illegal is morally wrong and doing so warrants punishment by virtue of its immorality. Sections two and three explore and critique the arguments for criminalising the possession, production and trafficking of drugs. Section four offers reflections on how drug policy in the UK and further afield can and should be modified to avert the ethical issues arising from a criminal justice focused approach.

## The immorality of (some) drugs

For some, the impetus for punitive drug policy seemingly emanates from the deontological idea that there is something morally wrong about using those drugs that are illegal and that doing so warrants punishment [6]. The meaning ascribed to ‘drug’, in the illicit sense, which fuels drug policy discourse and the paradigm of the war on drugs [7] encompasses a vast range of substances with different effects, used by different groups in different circumstances that have as much in common with each other as they do with many legal substances. In fact, the only defining characteristic shared by those drugs that are illegal, distinguishing them from those that are not, such as alcohol, tobacco or coffee, is precisely that they are illegal. This generates a circular argument: illegal drugs are immoral because they are illegal, and they are illegal because they are immoral. It is this circularity that unfortunately means that the position is as logically unassailable for those who hold it as it is nonsensical for those who do not.

Proponents of the view that drug use is immoral may claim that its immorality stems from its potential to cause harm to a consumer. However, it is not obviously correct to say that the potential for an act to cause harm necessarily makes it immoral. Choosing to go skiing or lighting a campfire, for example, could result in an injury or a burn; however, these acts would not be afforded moral status by virtue of their harming potential alone. On the other hand, forcing someone with no training to ski down a steep mountain or setting them on fire before doing so is clearly ethically problematic. If it is the agency of the subject at risk of suffering harm as a result of an act that determines the morality of that act, then it is not immoral for somebody to voluntarily expose themselves to the risk of harm by using a drug. Contrary to this position, it can be argued that adults have a moral right to do what they want to do to their own bodies, which would include using drugs for recreational purposes [8]. Even if it was accepted that it was immoral for subjects to put themselves in harm’s way, and that this should determine the legal status of different drugs, this is not reflected in current policy as the legal classification of different drugs is not representative of the relative levels of harm that they are responsible for [9]. If this position was accepted, there would be profound consequences, not only in terms of the legal status of alcohol and tobacco, but also that of unhealthy foods, extreme sports and driving.

A more compelling argument would be to suggest that buying illegal drugs is morally wrong because it provides funding to criminal organisations thereby facilitating other criminal activities that cause harm to third parties. However, this argument once again leads to circularity as the relationship is contingent on contemporary policy: possessing a drug is illegal because buying it is morally wrong; buying it is morally wrong because it provides funding for organised criminal gangs; it provides funding for organised criminal gangs because they control the market for that drug; and they control the market for that drug because it is illegal to possess it.

The idea that the use of some drugs is immoral, and the war on drugs that emanates from this view is ideological; that is, it can be characterised by a configuration of power leading to the imposition of a set of ideas, which give some particular interests the appearance of being universal [10]. It is not a natural or inevitable state of affairs that the consumption of particular substances, such as coffee or alcohol is widely accepted, or even encouraged while the consumption of others, such as amphetamines or cannabis apparently justifies stigmatisation and punishment. It is only through the influence of powerful historical actors who served to benefit from the propagation of this view that it is now so widely accepted [11] and it is far from the case that any benefits from a punitive approach to drugs are shared universally. Without a sustained exertion of power, which is most clearly apparent in the enforcement of punitive drug laws, the incoherence of these distinctions would be more plainly obvious to those subject to that power, as would the dearth of beneficial consequences from the criminalisation of drugs. To divert attention from the incoherence of ideological viewpoints, proponents can direct intent focus on the specifics of the subject matter to inspire an emotive response [10]. In the case of illicit drugs, this often involves highlighting the harm that drug use causes, which indeed may be profound in some cases. However, as previously noted, potential to cause harm does not in itself obviously imbue an act with moral status and this view does not give credence to a moral distinction between harmful illicit drug use and harmful licit drug use or other potentially harmful activities.

These deontological arguments based on circular reasoning and ideology are not a sufficiently rigorous foundation upon which to base policy decisions. Accordingly, the ethical analysis of drug policy which follows is undertaken primarily through a consequentialist lens; that is, does it reduce harm? However, a further deontological argument will be examined regarding the use of criminal sanctions to deter drug use, which is more robust than those posed against drug use itself.

## Criminalising the possession of drugs

When exercising punitive drug laws the use of force may lead to physical and psychological harm; contact with the criminal justice system is associated with a host of health and social inequalities which may be exacerbated by prosecution [12], and if leading to the deprivation of liberty, this is inherently harmful to the individual who is prevented from doing what they want to do. For this approach to be morally justified in consequentialist terms, it would need to prevent more harm than it causes. Proponents might claim that it results in a net reduction in harm to those being punished as it deters them from using drugs in the future. However, there is not convincing evidence that this is the case and even incarceration is not a reliable deterrent as more than one in four prisoners surveyed in the UK reported drug use in prison [13].

Regardless, data from the UK suggest that most people who take illicit drugs do not do so regularly [14] and as risk is cumulative, consumption and harm tend to be correlated [15]. Therefore, as Professor David Nutt highlights in the film, for the vast majority who use drugs, the negative impact of a criminal record would be much more significant than the negative impacts of continued infrequent drug use. For those who use drugs more frequently and problematically who are at the greatest risk of harm from doing so, use often develops in the context of adverse childhood experiences [16] and socioeconomic deprivation [17]. This is highlighted by Andria Efthimiou, who has first-hand experience of heroin use: “I was obviously reacting to … a very difficult childhood of illness that nearly killed me many times; difficult family circumstances, socially, economically; stressed out mother; absent father; and drugs were a great comfort so, having another punisher as it were with the police … what’s the point?” These antecedents of other health and social disadvantages are only exacerbated by contact with the criminal justice system and a criminal record.

Alternatively, proponents of punitive drug laws might argue that the harm they cause to individuals is justified by a net reduction in harm in society overall as others are deterred from drug use. Four challenges to this position follow: first, a direct empirical rebuttal; second, a consequentialist challenge related to the unintended negative impacts of criminalising possession; third, a deontological challenge in regard to the ethically problematic nature of using humans as a means to an end; and fourth, a procedural challenge highlighting the inequitable application of punitive drug laws.

First, there is no clear association between drug policy liberality and drug use prevalence, either contemporaneously across different countries [18] or subsequently in countries that have changed their drug policy [19, 20]. Although it is feasible that punitive laws might reduce drug use in some settings, it is not a necessary condition of doing so as in Portugal the use of some drugs has continued to decrease after possession for personal use was decriminalised [21].

Second, the application of punitive drug laws may encourage behaviours that increase the risk of harm in the wider drug taking population. Fear of punishment might result in people using drugs in more secretive and riskier ways, for example by taking larger amounts before leaving the house or taking drugs that they bought hastily without examining them [22]. In addition, they may not as readily engage with harm reduction or treatment services, which would otherwise have mitigated the risks that they are exposed to [23].

Third, even if there was convincing evidence that the punishment of people who use drugs deterred wider use and did not have unintended negative impacts, it is still a morally problematic approach as illustrated by an event in Voltaire’s *Candide.* The eponymous protagonist refuses to step foot on English soil after witnessing an admiral being ceremoniously shot in the head. Upon asking why the admiral was executed, the characters are told “in this country we find it pays to shoot an admiral from time to time to encourage the others” [24]. Voltaire wrote *Candide* to confront the position of Enlightenment philosophers who argued that everything happens for a reason and that the world is truly as perfect as it would need to be to vindicate their belief in an omnipotent, benevolent god. Contrary to this, Voltaire accused the world of being “a senseless and detestable piece of work” typified by the profound injustice of the execution of the admiral and the rationale that led to it. This is the same rationale that persists in contemporary drug policy: that it is fine to use an individual as a means to an end by making an example of them *pour encourager les autres* (to encourage the others). When explicitly stated as such, this approach is clearly incompatible with contemporary public health ethical guidelines [25]; the ‘fundamental British value’ of ‘individual liberty’ taught in schools as directed by the same government that bolsters a criminal justice approach to drugs [26]; and the writings of the philosophers who laid the foundations of European political thought, such as Immanuel Kant and John Stuart Mill, who decried the instrumentalisation of human beings [27, 28].

Finally, even if punitive drug laws deterred drug use, had no unintended negative consequences, and were otherwise morally justifiable, they are not applied equitably as members of some ethnic minority communities are punished for the possession of drugs disproportionately compared to the amount that they use drugs. This is notably the case in the USA [29] but also in the UK [30], persisting as a face of prejudice in a world acutely sensitised to the shadow of racial inequality. If there were benefits from a criminal justice approach to drugs, it would still be ethically problematic that these benefits were contingent on causing harm that was primarily shouldered by specific groups as determined by the colour of their skin.

## Criminalising the production and trafficking of drugs

In deontological terms, punishing the production and distribution of harmful drugs might seem more ethically tenable than punishing the possession of drugs for personal consumption. However, on closer examination this too is not straightforward as it is not always clear whether drug market actors are more accurately characterised as perpetrators of crime or victims of extenuating circumstances. In the UK, exploited children and vulnerable adults play a prominent role in the recently identified county-lines drug market model [31], and internationally, marginalised and deprived communities face significant pressures, financial and otherwise, to produce drugs [32]. In addition, as was the case when distinguishing between the consumption of legal drugs such as coffee and alcohol and illegal drugs such as amphetamines and cannabis, the distinctions between producing them are nominally legal, beyond which there is not a clear ethical difference. If it is the potentially harmful nature of a product that warrants laws being enacted against its production, then the production of alcohol, tobacco, refined sugar, and cars should similarly be criminalised.

From a consequentialist perspective, proponents of the war on drugs might claim that it reduces drug-related harm as the seizure and destruction of drugs prevents their consumption and the punishment of producers and traffickers deters others from entering the market thereby further reducing drug availability. Four consequentialist challenges to this position follow: first, a direct empirical rebuttal; second, related to the unintended corollary of increasing innovation; third, highlighting the harm caused to third parties; and fourth, highlighting the preclusion of more refined regulation of the market.

First, as former undercover police officer Neil Woods highlights in the film, the ninth principle of British policing states that the test of police efficiency is not evidence of police action, but the absence of crime [33]. If the global illicit drug market is considered in criminal terms, domestic and international policing efforts have categorically failed this test. Although vast amounts of money and effort have been devoted to combating the illicit drug trade, the market continues to grow [34] with the short-term impacts of interdiction proving as unsustainable as decapitating one head of the proverbial hydra. The astronomical profit margins available to drug traffickers mean that the cost of drug seizures can easily be absorbed as a ‘tax’ on their operations [35] and marginalised drug producing communities can be incentivised to continue production in spite of efforts to deter them from doing so [32].

Second, efforts to stop the production and distribution of drugs promote innovation, which can exacerbate and lead to new types of harm. For example, new drugs are developed to circumvent existing detection methods and legislation [36]; in the last 2 decades, more than 670 new psychoactive substances have appeared on the European drug market [37], for most of which very little is known about in terms of their health impacts or how to mitigate them [38]. And new means of distribution are devised to avoid enforcement efforts; in the case of the darknet, this has made drugs more readily available [39]; and in the case of the UK county lines phenomenon, this has promoted new forms of criminality and exploitation [31].

Third, in some cases, efforts to combat the drug trade can cause harm to third parties as communities and the environment become collateral damage caught in the crossfire of the war on drugs. In Colombia, for example, swathes of the country have been fumigated to destroy coca crops [40]; a practice some commentators have argued was in contravention of international humanitarian law [41]. Fumigation was stopped in 2015 after the World Health Organisation declared that the chemicals being used were probably carcinogenic [42]; however, it looks likely to recommence in the foreseeable future following pressure from the Trump administration [43].

Finally, the illegality of drug production precludes governmental regulation of the market or enforcement of standards of production to reduce harm. Variable drug purity [44] and the adulteration of drugs with stronger and more harmful substances, particularly the adulteration of heroin with fentanyl analogues in the USA [45], have been identified as key factors contributing to increasing drug-related death rates. In addition, the government cannot financially regulate an illegal market. In 2017, in the European Union alone, the illegal drug market was estimated to be worth between 26 and 34 billion euros [46]. This money, which would otherwise need to be accounted for and could be taxed, may be used to fund harmful activities, including other forms of organised crime and exploitation [47] and potentially terrorism [48].

## Ethical imperatives for drug policy

There is increasing political and academic support for countries to follow in the footsteps of Portugal and decriminalise the possession of drugs for personal use. This includes recommendations from the United Nations Chief Executives Board for Coordination [49], a 2019 UK House of Commons Select Committee on Drug Policy [50], the Royal Society of Public Health [51], the Canadian Association of Chiefs of Police [52] and the Lancet Commission on Drug Policy and Health [23]. Thus far, however, in the UK at least there seems to be little impetus for change.

Although the decriminalisation of the possession of drugs would be a step in the right direction, it does not confront the problems related to the unregulated nature of the criminal drug market. The legal regulation of all drugs would be more congruent, not only with the approach taken with drugs that are currently legal including alcohol and tobacco, but also with other products capable of causing harm, such as refined sugar and machinery. This is not to suggest that current models of regulation for legal products are necessarily correct and research on the commercial determinants of health highlights the need to approach questions of regulation extremely carefully [53]. A future in which all drugs are legally regulated is difficult to imagine; however, the prospect becomes more palatable when considering that opioid substitution and heroin-assisted therapy are essentially highly regulated markets for drugs, with convincing evidence in their favour that they reduce harm to individuals and wider society following reductions in acquisitive crime [54, 55].

The legal status of drugs should not be the crux of the drug policy debate; rather, the key consideration should be how to mitigate the harm they cause, which crucially means minimising the unbridled opportunity for drug market actors to profit from their sale. Neoliberal tendencies can be seen in the marketing practices of both the legal and illegal markets for drugs, which disregard the health and well-being of consumers for want of profit [31, 53]. Close regulation is required to ensure that if it was possible to make *any* profit from drugs, this is subsidiary to reducing the risk of harm and the prevalence of problematic use and dependence. This would include exerting control over how drugs are produced and who can buy them, where, when and with what caveats, as well as prohibiting marketing intended to widen the market. If the intention of regulating the drug market was to reduce the risk of harm and problematic drug use, this would clearly not be compatible with the institution of a free market for drugs, or for example, adverts for cocaine at the cinema. Equally, however, it does not necessarily mean that all those drugs that are currently illegal should only be accessible with a prescription as methadone is in the case of opioid substitution therapy. As one interviewee said in confidence, the key question is not *whether* illicit drugs should be legally regulated, but *how*. The regulation of different drugs should reflect the not so subtle differences between them; however, the global push to develop policies along the lines of the Psychoactive Substances Act in the UK, which prohibits the sale of any substances nebulously defined as ‘psychoactive’ aside from those arbitrarily exempted [56], further homogenises the management of a plethora of substances, which are defined by their granularity.

While a significant reprioritisation of drug policy in the UK is unlikely in the foreseeable future, more could be done to reduce the burden of drug-related harm without a drastic change in legislation. There is convincing evidence that drug treatment and harm reduction interventions including needle and syringe programmes are cost-effective investments that not only reduce harm, but also can lead to savings across many health, social and criminal justice services [57, 58]. Despite this, drug treatment budgets in the UK have decreased by nearly 30% in recent years [50], and although numbers in treatment increased slightly in 2018/19, this follows a consistent fall since 2013 [59].

The harm reduction movement, which promotes the provision of interventions that reduce the risk that people are exposed to when they choose to use drugs, while admitting that it is not possible to eliminate all drug use [60] offers a practical conception of consequentialist ethical theory [6]. However, unfortunately, the concept of harm reduction is misted in controversy, which seems to stem from the flawed position tackled in section one of this article: that the use of some arbitrarily defined substances is immoral. Alternatively, critics might be allowing for the perfect to be the enemy of the good by holding out for the cessation of all drug use, which induction would suggest is an unrealistic goal judging by the ineffectual decades spent waging the war on drugs. Relatively minor tweaks to policy would allow the provision of other harm reduction interventions with promising evidence in their favour, which are currently prohibited under the auspices of UK legislation.  For example, the Home Office has repeatedly refused calls to allow the provision of crack pipes by drug treatment services, which would be particularly pertinent in the current climate to minimise the transmission of COVID-19 as well as providing a means of engagement with people who use crack cocaine [61]. And despite the successful implementation of drug consumption rooms in other European countries [62], multiple calls to allow them to be opened in the UK have been rejected [63].

## Conclusion

None of these measures—the harm reduction movement, decriminalisation of the possession of drugs, or the regulation of the drug market—is a panacea, and even together they would not eliminate drug-related harm. However, neither will an ideological war on drugs, which is itself directly and indirectly responsible for incalculable harm to the significant proportion of the population who use drugs and to wider society. Some level of drug use and drug-related harm is as inevitable in the future as it has been present for millennia. Hopefully, one day this will be accepted by policymakers, and the vast resources spent waging the war on drugs will be redirected to reducing harm, rather than propagating it. Not only will the rights of those who use drugs need to be taken into account, but also the rights of the marginalised communities compelled to produce and distribute them if there is any hope of realising the optimistic future for all outlined by the United Nations Sustainable Development Goals.

The magnitude of the cultural gestalt switch and the level of international collaboration required for this to happen cannot be underestimated, and a future in which the war on drugs has ended is far from being realised. However, the COVID-19 pandemic has demonstrated that unprecedented policy change is possible in the face of overwhelming need. Although for most, the need for a change in drug policy is not as immediately tangible, from the perspective of those affected by the war on drugs, an overwhelming need is exactly what is faced. It should once again be stressed, however, that although rapid change is indicated in light of the many ethical problems arising from contemporary drug policy, extremely careful planning is required to mitigate the risk of unintended negative consequences, particularly in terms of the potential influence of actors who may wish to profit from the market. And, as the Lancet Commission on the legal determinants of health highlights, *ongoing* analysis is needed to ascertain how the law affects health, with the evaluation of new legislation, and consideration of its revision or repealment being as important, if not more so, than its drafting and enactment [64].

Although it is seemingly unlikely that the UK will spearhead a global rationalisation of drug policy, it is not beyond the realms of possibility following the sensible conclusions of the 2019 House of Commons Select Committee on Drug Policy [50]. Their initial report concluded that “UK drugs policy is failing” and among other things highlighted the potential benefits of decriminalising the possession of drugs, changing legislation to allow the opening of drug consumption rooms and increasing the provision of harm reduction interventions that are not widely available in the UK such as drug checking services and heroin assisted therapy.

For political actors to gain the mandate for change, however, it is necessary for the electorate to have a greater understanding of the intricacies of the issue, immeasurably more complex than a metaphorical understanding of drugs as an enemy that needs to be fought. Perhaps this is unrealistically optimistic; however, nothing has been achieved without optimism, and it is hoped that this short film might cause at least a few to give pause and reflect.

## Link to film

The short film *Putting UK Drug Policy into Focus* is available at the following link. It is indended to be used for educational purposes and as a tool for engaging with the public, policymakers and other professional groups. No permission is required to screen or share the film. A shorter version, and a recording of the webinar at which the film was launched at the 2020 European Harm Reduction Conference are available on the YouTube channel 'Drug Policy in Focus'. https://www.drugscience.org.uk/uk-drug-policy-focus/.

## Data Availability

Not applicable.

## Abbreviations

COVID-19: Coronavirus Disease 2019
UK: United Kingdom
USA: United States of America
